# Post-infarct ventricular tachycardia masquerading as supraventricular tachycardia: Using 3-dimensional mapping to delineate the circuit between scar and His-Purkinje system

**DOI:** 10.1016/j.hrcr.2024.11.020

**Published:** 2024-12-03

**Authors:** Justin Chiong, Mark T. Mills, Peter Calvert, Claire O’Neill, Vishal Luther

**Affiliations:** 1 Department of Cardiology, Liverpool Heart and Chest Hospital NHS Foundation Trust, Liverpool, United Kingdom; 2 Liverpool Centre for Cardiovascular Science, University of Liverpool, Liverpool John Moores University and Liverpool Heart and Chest Hospital, Liverpool, United Kingdom; 3 Biosense Webster, Johnson and Johnson Medical Ltd, Wokingham, Berkshire, United Kingdom

**Keywords:** Ventricular tachycardia, Ripple mapping, Post-infarct scar, Cardiac resynchronization therapy, Arrhythmia mechanisms

**Key Teaching Points**

• Post-infarct scar–related ventricular tachycardia can rarely present as a narrower complex tachycardia mimicking supraventricular tachycardia.
• This can occur in patients with remote anterior myocardial infarction with left ventricular basal septal scarring.
• Using detailed 3-dimensional mapping, we have shown that the His/left bundle branch/Purkinje system lie adjacent to the diastolic pathway/exit within septal scar.
• Targeted ablation of the diastolic pathway is highly effective in eliminating this ventricular tachycardia circuit.

## Introduction

Ventricular tachycardia (VT) typically manifests with a broad QRS complex on the surface electrocardiogram (ECG), as the re-entrant circuit involves slow conduction around lines of block within ventricular myocardium.^1^ VT can manifest deceptive features, such as a sharp intrinsicoid deflection or a narrower QRS, rendering diagnosis challenging.^2^ Such cases can occur in circuits involving the His-Purkinje system. In patients with prior myocardial infarction (MI), VT may rarely present with a narrower QRS complex, and the underlying mechanisms in such cases remain elusive.

Catheter ablation targeting areas of slow conduction around lines of block can eliminate post-infarct VT. Ablation is dependent on 3-dimensional (3D) activation mapping of the electrogram (EGM) signatures of slow conduction (ie, diastolic signal during VT and late potentials in sinus rhythm) to delineate the re-entrant circuit. On the CARTO platform (Biosense Webster, Wokingham, Berkshire), this includes ripple mapping, which displays the entire EGM as a moving white bar on the mapped surface anatomy, and wavefront discontinuity line (WADL) mapping, which highlights adjacent areas with prespecified differences in local activation time (LAT) as white lines on the map. The application of these activation mapping techniques during VT ablation has been described previously,^3^^,^^4^ and ripple mapping has recently been used to characterize the conduction system by distinguishing His-Purkinje EGMs from surrounding myocardial signal.^5^

In this series, we used 3D activation mapping to describe 3 rare cases of patients undergoing ablation of post-infarct VT, each presenting with deceptive surface ECG morphologies that could be misinterpreted as supraventricular tachycardia (SVT). All patients provided written informed consent for their anonymized case material to be considered for publication.

## Case reports

### Case 1

**Figure 1:**
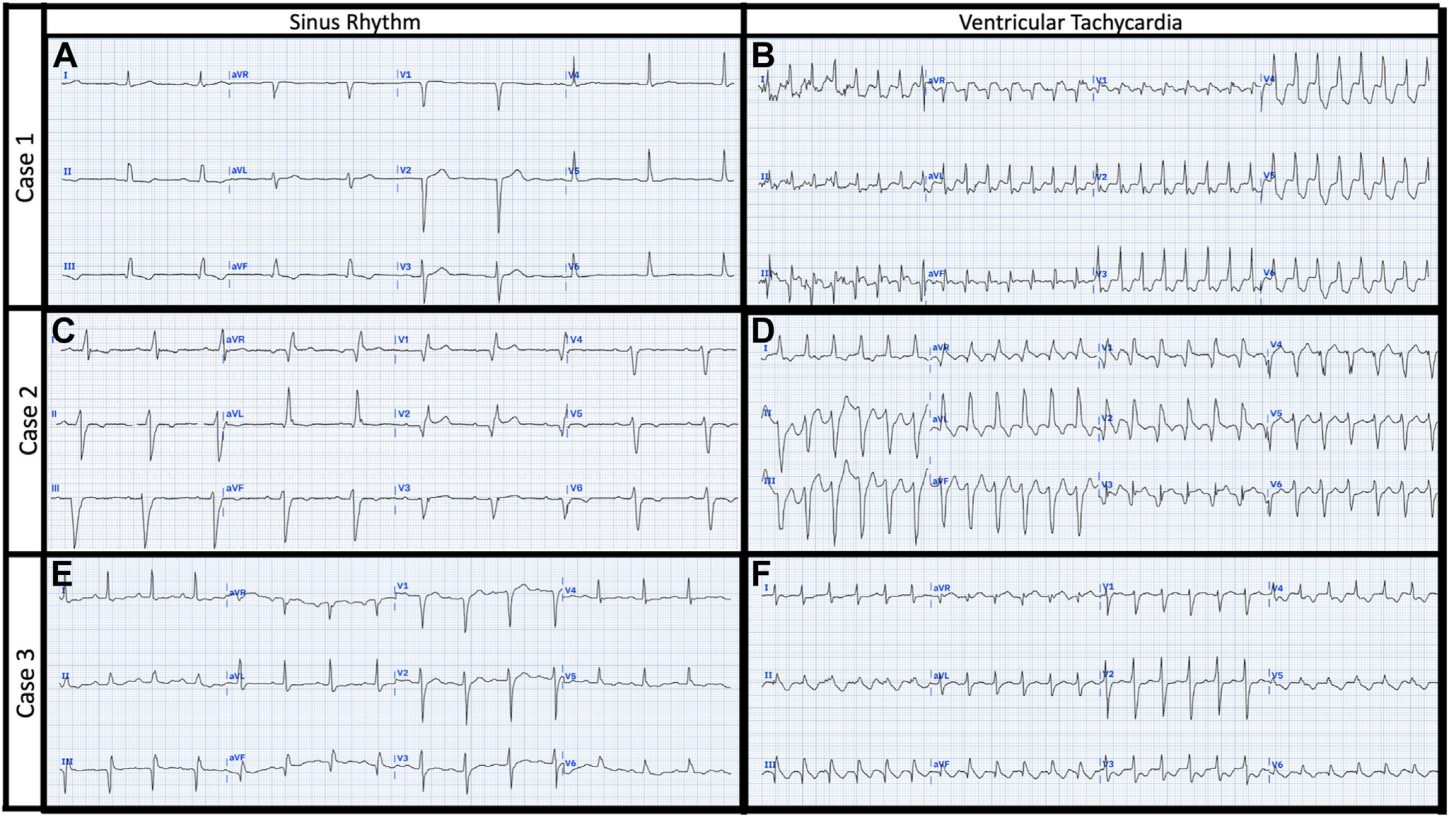
The 12-lead surface electrocardiograms (ECG) for cases 1, 2, and 3. **A, C, E:** Sinus rhythm. **B, D, F:** Ventricular tachycardia. See case text for detailed description of each ECG.

The first patient was a 79-year-old man with a history of anterior MI (2004) with coronary artery bypass grafting and severely impaired left ventricular (LV) systolic function (ejection fraction [EF] <35%). A 12-lead ECG revealed sinus bradycardia with a narrow QRS (112 milliseconds) and anteroseptal Q waves (Figure 1A). Echocardiography revealed a thin and akinetic basal-mid septal LV wall. A secondary prevention dual-chamber implantable cardioverter-defibrillator (ICD) was implanted previously after an earlier admission with VT, and a subsequent admission with VT storm was managed with amiodarone. Catheter ablation was planned to prevent further VT storm and facilitate discontinuation of amiodarone.

The 12-lead ECG in VT demonstrated a slightly more leftward QRS axis, as well as early R’ waves in V1–3; however, the QRS complex remained narrow (119 milliseconds), which could mislead the observer into considering SVT as the most likely diagnosis (Figure 1B). This rhythm was, however, confirmed as VT on ICD interrogation with atrioventricular dissociation.

**Figure 2:**
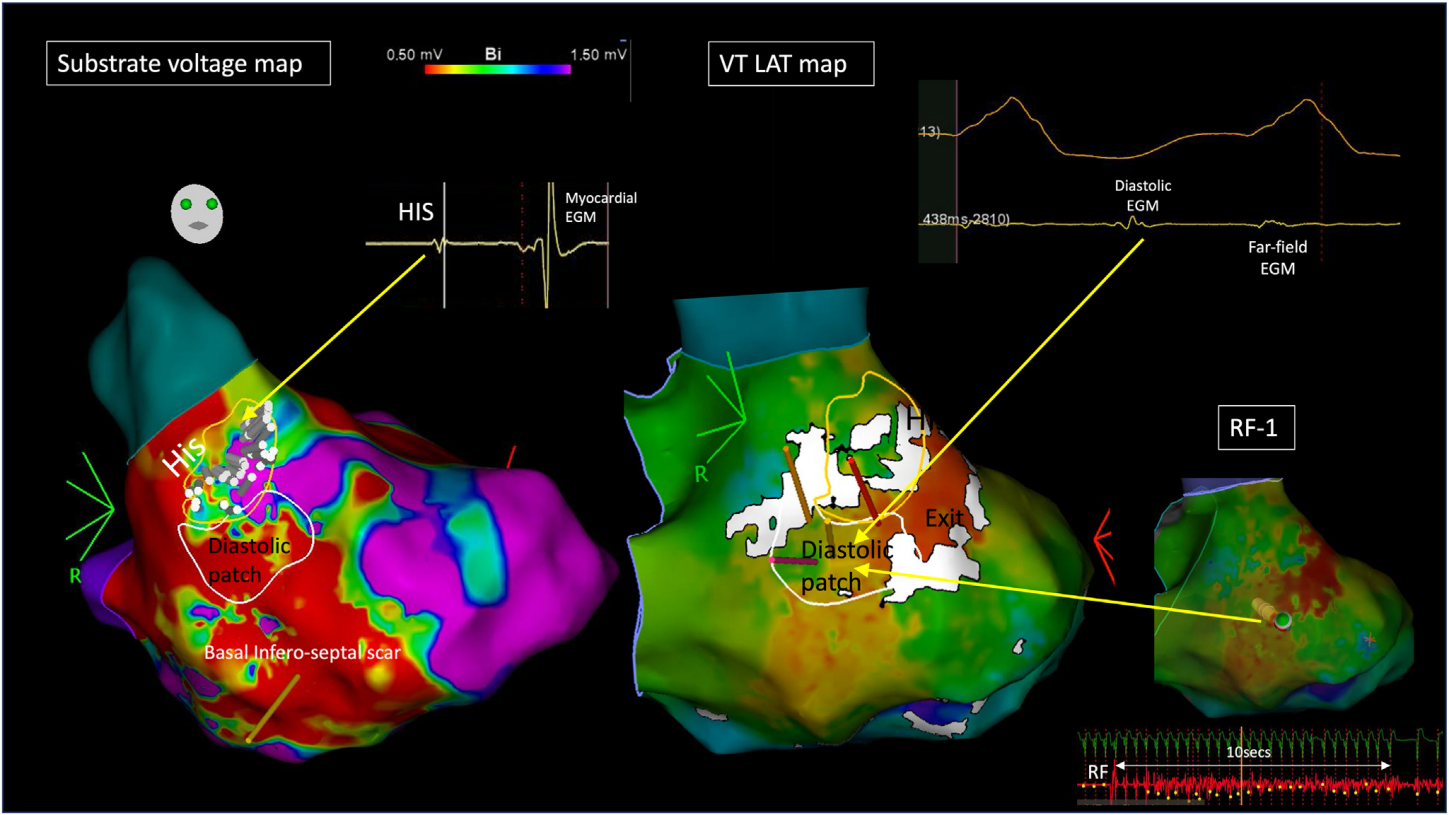
Case 1. *Left:* Left ventricular CARTO (Biosense Webster, Wokingham, Berkshire) bipolar voltage (0.5–1.5 mV) map collected in atrial paced rhythm. His patch highlighted by ripple map encircled in *yellow design line*. *Right:* local activation time (LAT) map in ventricular tachycardia (VT) suggesting focal activation (*red*) adjacent to the His patch. Extended early meets late set to 25% with *white* wavefront discontinuity lines (WADLs) highlighting adjacent areas with LAT difference >25% the mapped tachycardia cycle length, with WADL noted between exit site and His. Patch of mid-diastolic electrograms (EGMs) highlighted by ripple map encircled in *white design line*. The His, diastolic patch, and VT exit are all adjacent. *White design* overlaid on the left substrate map. *Top right:* Example of diastolic EGM. *Bottom right:* Ablation catheter at mid-diastolic site with VT termination in 11 seconds.

Catheter ablation was performed under conscious sedation to maximize VT inducibility. The LV was mapped with CARTO using an Octaray mapping catheter (Biosense Webster) via a transseptal approach. Substrate mapping was performed in an atrial paced rhythm to delineate the conduction system. Scar (defined as bipolar tissue voltage <0.5 mV) was observed across the basal to mid inferoseptum, overlying the presumptive anatomic location of the left posterior fascicle. The remaining LV cavity was of normal voltage (>1.5 mV). An area of His bundle potentials was visually differentiated from adjacent myocardial activation on the ripple map (Figure 2 and Supplemental Video 1). The “show bars above” function was set at 0.07 mV to avoid noise signal obscuring the identification of conduction system EGMs on the map. Subsequently, a nonclinical VT (QRS 160 milliseconds)—broader but with similar axis to intrinsic conduction—was easily induced and well-tolerated, facilitating mapping (cycle length [CL] 440 milliseconds). WADL was to highlight adjacent areas with EGM timing differences >20% of the mapped CL. An LAT map captured only 213 milliseconds of CL in VT and suggested focal exit directly adjacent to the His with WADL in between. Importantly, close inspection of the ripple map revealed a patch of mid-diastolic potentials immediately beneath the His, adjacent to the exit site. The first radiofrequency application (40 W, irrigated) at the diastolic patch resulted in slowing and termination of the VT in 11 seconds. Further substrate ablation (27 lesions, 40 W, 30 seconds) was delivered around the low-voltage area, preserving native conduction in the absence of biventricular pacing support and rendering the patient noninducible. The sinus rhythm QRS width remained <120 milliseconds at procedure end. At 3-month follow-up, he remained VT-free off amiodarone.

### Case 2

The second patient was a 61-year-old man scheduled for inpatient catheter ablation after emergency department attendance for VT. He also had previous anterior MI and severely impaired LVEF (<35%), with septal akinesia. A 12-lead ECG in sinus rhythm (Figure 1C) revealed a QRS of 132 milliseconds with left anterior fascicular block and deep Q waves anteriorly, with loss of R-wave progression across the lateral precordial leads. A cardiac resynchronization therapy (CRT) defibrillator device had previously been implanted as primary prevention. A 12-lead ECG in tachycardia was of similar morphology and width (135 milliseconds) to sinus rhythm (Figure 1D)—initially thought to suggest SVT—although later confirmed as VT through ICD interrogation. This remained resistant to amiodarone.

**Figure 3:**
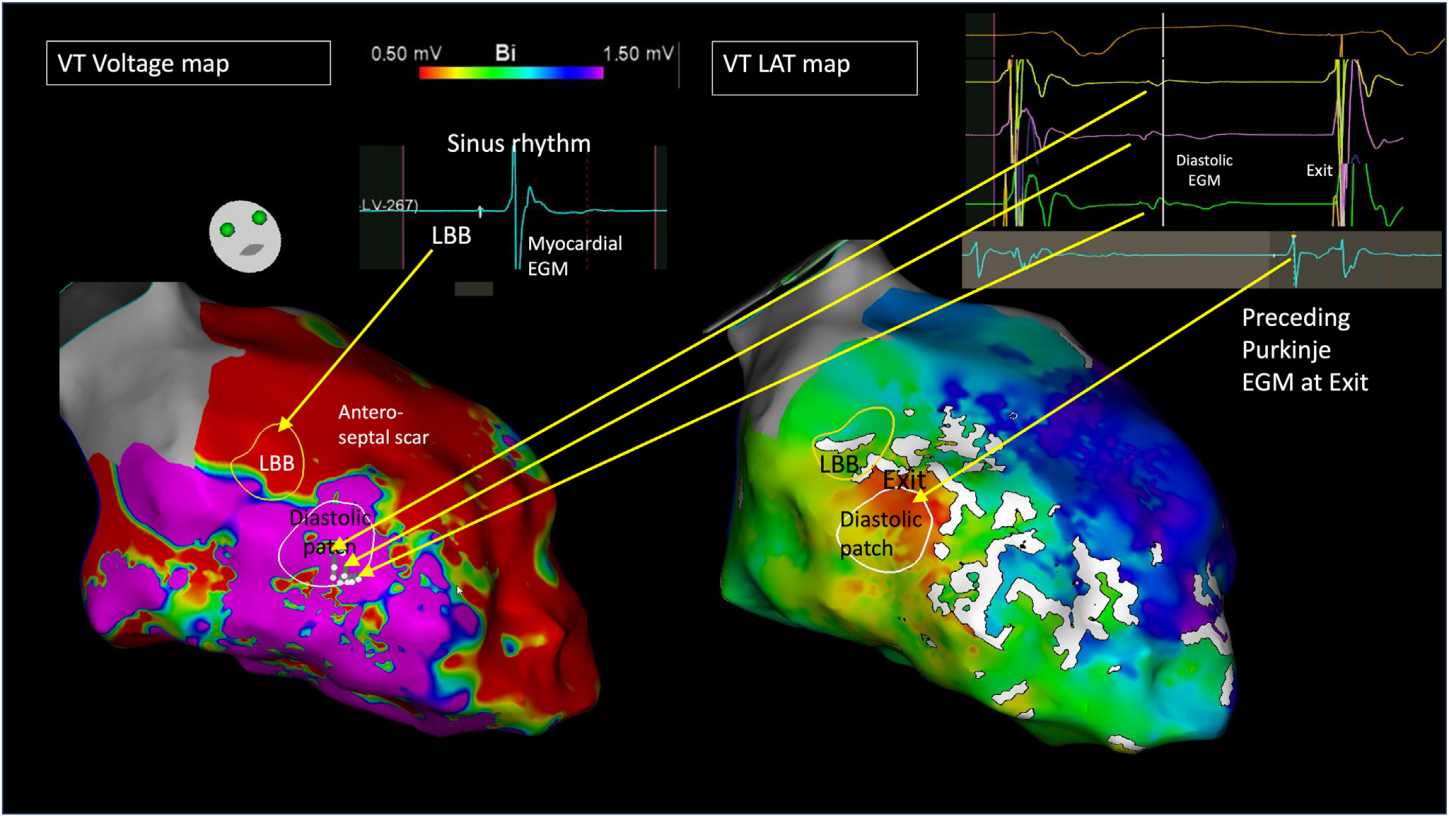
Case 2. *Left:* Left ventricular CARTO (Biosense Webster, Wokingham, Berkshire) bipolar voltage (0.5–1.5 mV) map collected in ventricular tachycardia (VT). Patch of diastolic electrograms (EGMs) highlighted by ripple map encircled in *white design line*. Left bundle branch (LBB) EGM mapped in intrinsic rhythm encircled in *yellow design line*. *Right:* Local activation time map in VT suggesting focal activation (*red*) adjacent to diastolic patch and LBB. Wavefront discontinuity line (WADL) noted between exit site and LBB. The LBB, diastolic patch, and VT exit are all adjacent. *Top right:* Example of diastolic EGM from diastolic patch and Purkinje-type EGM from focal exit.

Ablation was performed under conscious sedation. Clinical VT with a similar morphology and width to intrinsic conduction was readily inducible and well-tolerated, and the LV was mapped in VT via a transseptal approach with a Pentaray (Biosense Webster) catheter (Figure 3). Detailed substrate mapping was not possible due to incessant VT, but the location of the left bundle branch (LBB) was identified during momentary intrinsic conduction (containing no associated atrial EGM). Voltage mapping in slow VT (CL 435 milliseconds) revealed extensive septal low voltage extending from base to apex, overlying the anatomic location of the left anterior fascicle, in keeping with the resting ECG pattern. The remaining LV cavity was of normal voltage in VT. The LAT map collected only 197 milliseconds of activation in tachycardia and demonstrated focal exit with probable preceding Purkinje potential, directly beneath the LBB, with WADL in between. The Ripple map captured a patch of diastolic potentials, immediately beneath the LBB signals, and adjacent to the exit site. Ablation at the diastolic signal promptly terminated VT, rendering it noninducible. A total of 20 ablations lesions (40 W, 30 seconds) were delivered. The LBB was spared, with no change in QRS width or morphology. At 25-month follow-up, he remained VT-free off amiodarone.

### Case 3

The final patient was a 59-year-old man admitted with recurrent VT and scheduled for inpatient ablation. Similarly to cases 1 and 2, he had previous anterior MI (2007), severely impaired LVEF, and septal akinesia. A 12-lead ECG in sinus rhythm (Figure 1E) revealed a QRS of 125 milliseconds with nonspecific intraventricular conduction delay. A CRT defibrillator had previously been implanted as secondary prevention (LV lead implanted owing to the concomitant use of amiodarone and anticipated progression of conduction disease). The captured 12-lead ECG in tachycardia was almost identical to intrinsic conduction (QRS 128 milliseconds, Figure 1F) and initially interpreted as SVT by the admitting general physician, although VT was rapidly confirmed through ICD interrogation. VT was recurrent, despite amiodarone and mexiletine, with >500 episodes sensitive to antitachycardia pacing during an intercurrent COVID-19 illness.

**Figure 4:**
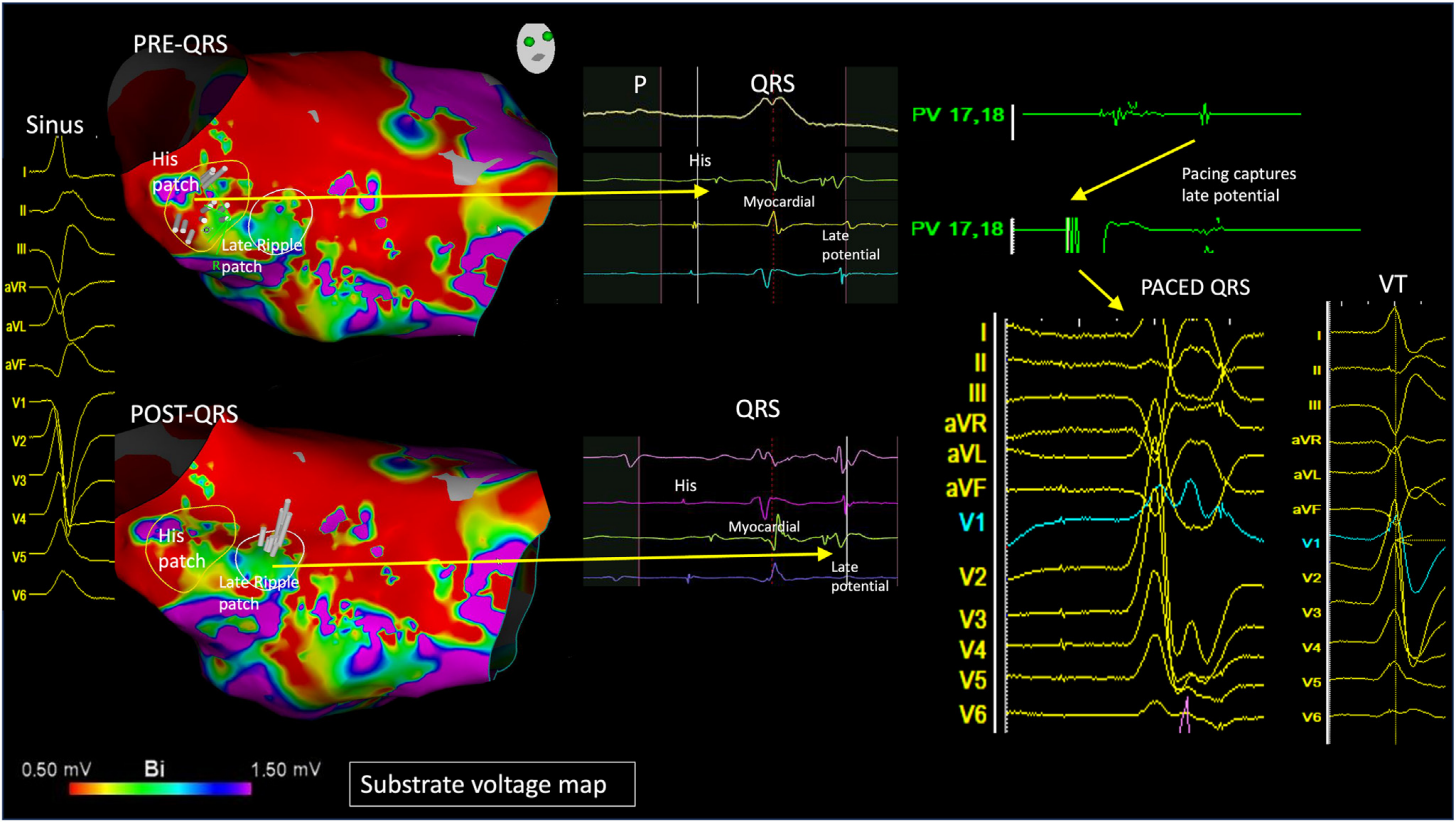
Case 3. *Far left:* Sinus QRS template. *Left:* Left ventricular CARTO (Biosense Webster, Wokingham, Berkshire) bipolar voltage (0.5–1.5 mV) map collected in atrial paced rhythm. His patch highlighted by ripple map encircled in *yellow design line*. Post QRS, a distinct patch of late ripple activation, encircled in *white design line*, seen beneath His patch. *Middle:* Electrogram examples of 3 distinct potentials (His, myocardial, and late activation). *Right:* Pacing captures the late potential and reveals a narrow QRS, similar to the clinical ventricular tachycardia.

Under conscious sedation, clinical VT was inducible but poorly tolerated. A substrate map of the LV was collected via a transseptal approach in an atrial paced rhythm using a Pentaray catheter. Areas of low voltage were noted across the basal to mid septum, including over the His, extending over the anatomic location of the left anterior fascicle. The His was visually differentiated from myocardial activation on the ripple map (Figure 4, Supplemental Video 2). Interestingly, around the vicinity of the His/LBB area, EGMs revealed 3 distinct potentials; the first was conduction system; the second was myocardial; and the third was a late potential of uncertain etiology. Pacing to capture the late potential revealed a narrow QRS, similar to clinical VT. The His was thought to be intimately involved in the circuit, and a decision was made to ablate the entire basal septal substrate, including this His (48 lesions, 25 minutes, 30 seconds at 50 W irrigated) and render the patient pacing-dependent, given the presence of a pre-existing CRT device. After this, VT was noninducible. At 24-month follow-up, he remained VT-free, off all anti-arrhythmic medication.

## Discussion

Post-infarct VT with QRS morphology similar to intrinsic conduction has been described rarely in the literature.^6^ We presented 3 cases in which patients underwent detailed activation mapping to delineate the mechanism of this unusual occurrence.

Notably, the 3 patients shared several characteristics: they had a history of remote anterior MI and severely impaired LV function, and their cardiac imaging revealed a thin and akinetic basal septum, presumably a result of occlusion of a septal branch of the left anterior descending artery during the infarct process.^7^ Detailed 3D voltage maps confirmed probable scar tissue (<0.5 mV) involving the LV basal septum, in proximity to the His/LBB, which may facilitate rapid conduction through the ventricles, manifesting as a narrower complex QRS and/or similar morphology to sinus rhythm on the 12-lead surface ECG.^8^

Purkinje fibers and ventricular muscle can both survive within healed infarcts and participate in such circuits.^9^ The Purkinje system is vulnerable to re-entry, as evident in fascicular VT.^10^ Click or tap here to enter text.In a series of 81 patients with post-infarct VT by Bogun and colleagues,^11^ 8 patients presented with QRS <145 milliseconds and all had evidence of septal scar. Purkinje potentials preceded the QRS complex in sinus rhythm at the corresponding VT exits sites. However, unlike in our series, VT morphology did not mimic the QRS pattern of intrinsic conduction as closely. This suggests that the circuit in our 3 cases may have exited more proximally within the conduction system, between the His and LBB.

Using ripple mapping, the proximal components of the conduction system (His/LBB) were readily apparent, situated adjacent to (cases 1 and 2) or within (case 3) the scar. VT was mapped in 2 patients, and the LAT map suggested focal activation from the exit site with a line of slow conduction/block (WADL) between it and the His/LBBB. Ripple mapping, which displays a white moving bar for every EGM deflection, highlighted a patch of low-amplitude endocardial diastolic EGMs, seen to border these conduction system EGMs. Yoshida and colleagues^12^ suggested such VTs may appear focal due to a deep intramural circuitry focus with endocardial breakthrough pointsClick or tap here to enter text., but cases 1 and 2 challenged this concept, as evident by endocardial diastolic activity identified using high-density mapping. In the study by Yoshida and colleagues, ablation at the exit failed to terminate VT, whereas ablation at the diastolic EGMs terminated VT in our cases.

**Figure 5:**
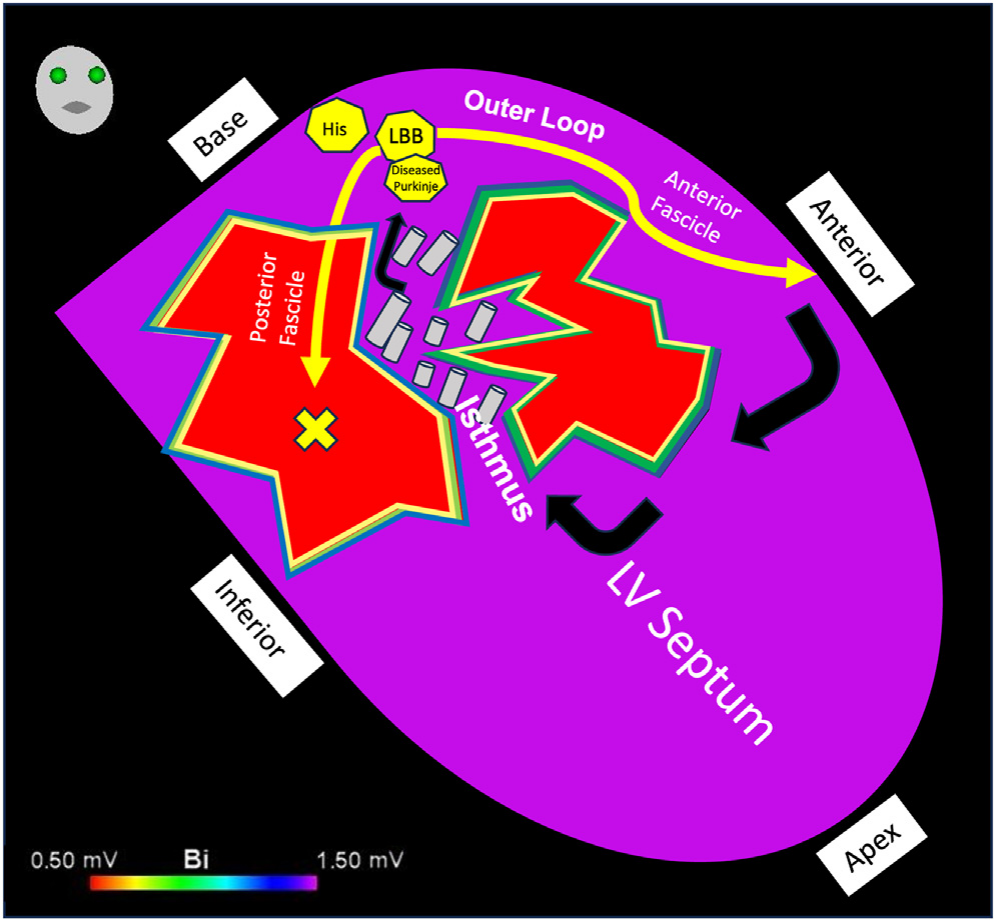
An illustration of the putative mechanism of narrower complex ventricular tachycardia: ripple bars traverse through a septal diastolic isthmus between areas of scar, before exiting adjacent to the His/left bundle branch/Purkinje system. LV, left ventricle.

The VT exit in cases 1 and 2 appeared to adjoin both the diastolic EGMs and His/LBB/Purkinje system. We hypothesized that early engagement of the diastolic pathway with the proximal left-sided conduction system facilitated this narrow QRS, as the VT circuit “piggybacked” onto the surviving conduction fibers. Despite their anatomic proximity, the interval between the diastolic EGMs and the exit is prolonged and may be consequent to latent activation crossing from basal myocardial tissue across the insulated His-Purkinje fibers, as highlighted by the presence of WADL. An illustration of this mechanism is presented in Figure 5. In case 3, the His/LBB was composed of 3 distinct EGMs, including conduction system; surrounding myocardium; and a third late potential, which we hypothesized represented either a split potential around a line of block or retrograde Purkinje activation. Our 3 cases share similarities with bundle-branch re-entrant VT, as there is intrinsic disease within the conduction system supporting re-entry in both mechanisms. However, bundle-branch re-entrant VT is a bundle-to-bundle circuit that is considered independent of scar/myocardial lines of block.

The amount of ablation required in each case was relatively limited in our cases (13 minutes, 10 minutes, and 25 minutes) compared with conventional post-infarct substrate ablation (typically approximately 30 minutes).^13^ In our series, medium-term outcomes after ablation were encouraging, with complete elimination of VT without anti-arrhythmic therapy at most recent follow-up. This contrasts with the nonischemic population with basal septal fibrosis, in which recurrence rates are high, despite ablation, suggesting that post-infarct critical sites are located within the basal endocardial surface, where the conduction system lies, as opposed to nonischemic intramural sites.^14^

The conventional indication for CRT therapy includes LVEF <35% and QRS >150 milliseconds. Guidelines do not recommend CRT in patients with a QRS <130 milliseconds. However, given our present observations, an argument can be made for patients with anterior MI and basal septal scar to be considered for CRT defibrillator in preference to ICD alone—especially if the device is implanted for secondary prevention—to facilitate potentially curative ablation of septal circuits, if they occur.

### Limitations

Our findings are based on 3 cases only. Given the application of high-density activation maps, entrainment mapping was not used in the diagnostic process.

## Conclusion

Post-infarct anteroseptal scar-related VT can masquerade as supraventricular arrhythmia, likely due to the close proximity of the VT exit, diastolic pathway, and His-Purkinje system, allowing the VT to potentially “piggyback” off the native conduction system.

## Disclosures

Dr. Luther has received speaker fees and a research grant in relation to ripple mapping; the rest of the authors have no conflicts of interest.
